# HHV-6 encoded small non-coding RNAs define an intermediate and early stage in viral reactivation

**DOI:** 10.1038/s41525-018-0064-5

**Published:** 2018-09-05

**Authors:** Bhupesh K. Prusty, Nitish Gulve, Suvagata Roy Chowdhury, Michael Schuster, Sebastian Strempel, Vincent Descamps, Thomas Rudel

**Affiliations:** 1 Biocenter, Chair of Microbiology, University of Würzburg, Würzburg, Germany; 2 CeMM Research Center for Molecular Medicine of the Austrian Academy of Sciences, Vienna, Austria; 3 Microsynth AG, Balgach, Switzerland; 4 Department of Dermatology, Bichat Hospital, APHP, Paris 7 University, Paris, France; 5 Helmholtz Institute for RNA-based Infection Research (HIRI), Würzburg, Germany; 6 Present Address: Institute for Virology and Immunobiology, Versbacher Str. 7, 97078 Würzburg, Germany

## Abstract

Human herpesvirus 6A and 6B frequently acquires latency. HHV-6 activation has been associated with various human diseases. Germ line inheritance of chromosomally integrated HHV-6 makes viral DNA-based analysis difficult for determination of early stages of viral activation. We characterized early stages of HHV-6 activation using high throughput transcriptomics studies and applied the results to understand virus activation under clinical conditions. Using a latent HHV-6A cell culture model in U2OS cells, we identified an early stage of viral reactivation, which we define as transactivation that is marked by transcription of several viral small non-coding RNAs (sncRNAs) in the absence of detectable increase in viral replication and proteome. Using deep sequencing approaches, we detected previously known as well as a new viral sncRNAs that characterized viral transactivation and differentiated it from latency. Here we show changes in human transcriptome upon viral transactivation that reflect multiple alterations in mitochondria-associated pathways, which was supported by observation of increased mitochondrial fragmentation in virus reactivated cells. Furthermore, we present here a unique clinical case of DIHS/DRESS associated death where HHV-6 sncRNA-U14 was abundantly detected throughout the body of the patient in the presence of low viral DNA. In this study, we have identified a unique and early stage of viral activation that is characterized by abundant transcription of viral sncRNAs, which can serve as an ideal biomarker under clinical conditions.

## Virology: a biomarker for HHV-6 reactivation

The human herpesvirus 6 (HHV-6) expresses high levels of small non-coding RNA (sncRNA) molecules early in its reactivation from latency. Bhupesh Prusty  from the University of Würzburg, Germany, and colleagues developed a laboratory system for studying HHV-6 infections in a human bone cancer cell line. They reawakened the virus with a drug stimulus and detected several sncRNAs but few other viral RNAs that might promote replication or protein production. They term this unique stage of the viral life cycle ‘transactivation’, and show that it alters both host and viral physiology. The authors also describe a teenage girl with high sncRNA levels in her blood who fell ill after an acne drug spurred the reactivation of a dormant HHV-6 infection. They thus argue that sncRNAs could serve as an early diagnostic indicator of HHV-6 reactivation.

## Introduction

Betaherpesviruses like human herpesvirus 6A (HHV-6A), HHV-6B and HHV-7 integrate into subtelomeric ends of human chromosomes and acquire latency.^1,2^ It is speculated that homologous recombination between telomeric repeat sequences allow viral integration as genetically altered herpesviruses lacking telomeric repeat sequences fail to integrate efficiently into human telomeres.^3,4^ Several HHV-6 latency associated genes have been described but functional significance of such gene products are not well understood. One such HHV-6 transcript, U94, has long been regarded as a latency-associated marker.^5,6^ However, recent studies have not found any significant role of U94 in viral integration or latency.^7^ Similar to viral latency, initiation of viral reactivation from latency that lays the foundation for further changes in viral and host cell fate is fairly unknown. An intermediate stage in viral latency that marks the beginning of viral reactivation was previously postulated.^8^ Further understanding of these initial steps of viral reactivation is important not only from viral life cycle point of view but also from host side as they can provide crucial information linking viral reactivation to human diseases.

Virus encoded small non-coding RNAs (sncRNAs) including microRNAs (miRNAs) play crucial role in establishment of viral infection, immune evasion as well as disease development.^9–11^ Similarly panel of host miRNAs also defines key steps during viral latency and viral activation.^10,12^ HCMV encoded sncRNAs like miR-UL148D and miR-UL112-1 allows successful viral latency.^13,14^ At the same time successful HCMV lytic infection depends upon downregulation of several human miRNAs including miR-200.^15,16^ Both HHV-6A and HHV-6B transcribes sncRNAs during productive viral infection.^17,18^ However, their transcription dynamics during viral latency and viral reactivation is not studied before. Hence we utilized a unique U2OS cell-derived latent HHV-6A cell culture model to understand viral reactivation and its impact on host cell physiology. In this study we have identified a unique expression pattern of virus-encoded small non-coding RNAs (sncRNAs) that clearly differentiates viral latency from reactivation. Furthermore, we provide clinical evidence showing abundance of these sncRNAs under clinical conditions of viral activation.

## Results

### Integrated viral genome is often lost during initial stages of viral reactivation

Understanding initial stages of HHV-6 reactivation strongly depends upon a robust cell line carrying homogeneous latent viral infections. To generate such a cell line, we utilized bacterial artificial chromosomes (BAC) derived^19^ HHV-6A viral particles carrying coding potentials for either recombinant green fluorescence protein (GFP)^20^ or red fluorescence protein (RFP) as these reporter cells are ideal for cell sorting and further selection of clonal population of homogeneous cells undergoing viral reactivation.

**Fig. 1:**
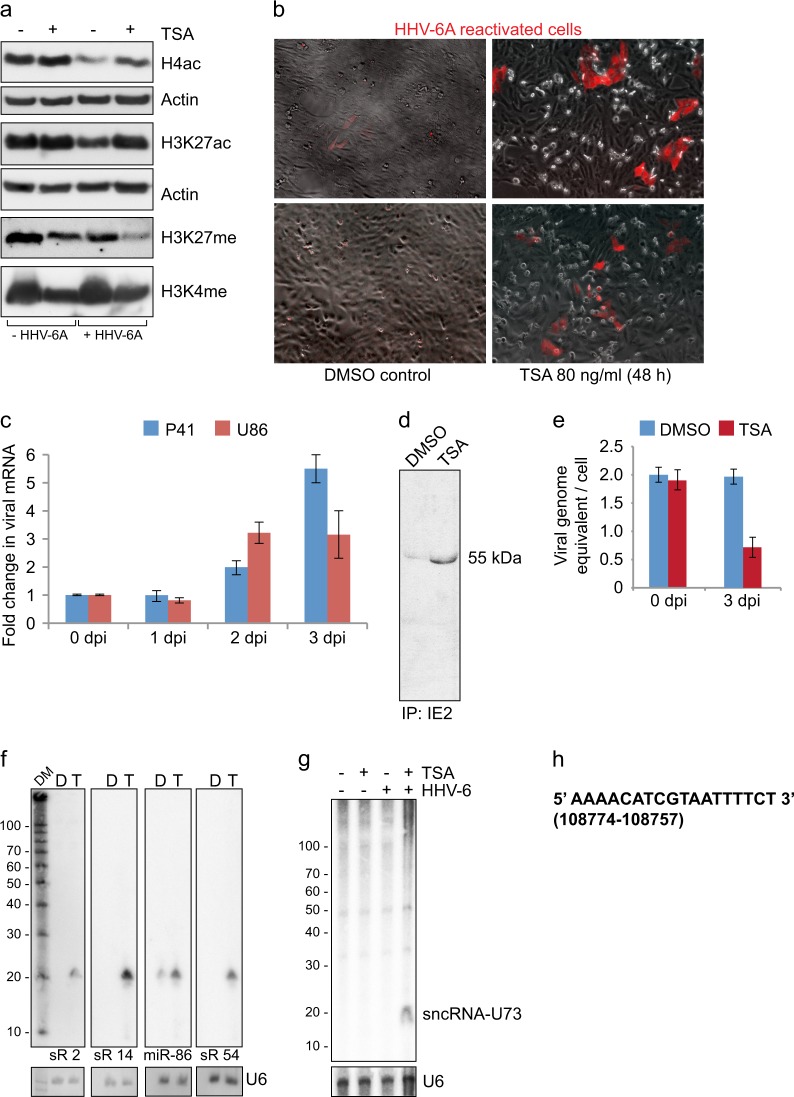
Characterization of HHV-6 reactivation. **a** Trichostatin A (TSA) induced histone acetylation in U2OS cells. TSA (80 ng/ml) was added to U2OS cell culture media for 24 h to induce histone acetylation. Total protein was extracted and used for immunoblotting. Histone H4 pan acetylation (H4ac), Histone 3 K27 acetylation (H3K27ac), Histone 3 K27 methylation (H3K27me) and Histone 3 K4 methylation (H3K4me) were studied using specific antibodies. Actin was used as loading control. U2OS cells without HHV-6 (-HHV-6A) were used as control. **b** TSA treatment induced expression of RFP in latent HHV-6A carrying U2OS cells. Microscopic evaluation was carried out for RFP expression in U2OS cells carrying latent HHV-6A. Cells were treated with DMSO in parallel as a solvent control. **c** TSA-induced HHV-6A reactivation was quantified by qRT-PCR analysis of two early viral transcripts (p41 and U86). U2OS cells carrying latent HHV-6A were treated with 80 ng/ml of TSA for three different time intervals. Total RNA was extracted and were used for cDNA synthesis and subsequent RT-PCR. Data represent the mean ± SEM of three independent experiments. dpi, days post infection. **d** Immunoprecipitation (IP) of HHV-6 IE2 protein was carried out to test immediate early protein synthesis. U2OS cells carrying latent HHV-6A were treated with DMSO or TSA for 2 days. Total cell lysates were extracted and used for immunopreciptation. A smaller ~ 55 kDa fraction of IE2 was detected in IP. **e** Viral DNA replication was studied by qPCR. U2OS cells carrying latent HHV-6A were treated with 80 ng/ml of TSA or DMSO for two different time intervals. Total genomic DNA was extracted and were used for qPCR analysis. Data represent the mean ± SEM of three independent experiments. dpi, days post infection. **f** Detection of several different HHV-6A encoded small non-coding RNAs by Northern hybridization. U2OS cells carrying latent HHV-6A were treated with 80 ng/ml of TSA (T) or DMSO (D) for 48 h. 10 μg of total RNA were separated on a denaturing Urea gel for Northern hybridization. Decade marker (DM) was used to verify sizes of identified RNA. Transcription of previously described small non-coding RNAs (labeled as sR) was tested using specific DNA probes. Human U6 RNA was used as loading control. **g** Detection of newly identified HHV-6A encoded sncRNA-U73 by Northern hybridization as described in figure f. **h** Sequence details of newly identified sncRNA-U73. Genomic location of the sncRNA-U73 is indicated as mapped to HHV-6A U1102 genome (Genebank X83413.2). All blots or gels (**a**, **d**, **f** and **g**) derive from the same experiment and they were processed in parallel

For the present study, we opted for human U2OS cells as viral reactivation is comparatively easier in these cells owing to their telomerase negative status because of which these cells prefer alternative lengthening of telomere (ALT) for maintenance of telomere length leading to increased telomeric-circle formation. Previous studies have corroborated the above-mentioned fact.^21^ To evaluate potential viral reactivation in these cells, we treated a homogeneous clonal population of U2OS cells carrying latent HHV-6A with a histone deacetylase (HDAC) inhibitor-Trichostatin A (TSA), which is known to activate latent HHV-6.^22^ HDAC inhibitors induce histone acetylation and reduce simultaneous histone methylation, which we verified by immunoblotting (Fig. 1a). Upon treatment with TSA at 80 ng/ml concentrations for 48 h, a pool of U2OS cells expressed the reporter RFP protein (Fig. 1b). In order to correlate the appearance of RFP signal with viral reactivation, we analyzed several different immediate early (IE) and early viral transcripts by qRT-PCR. The results revealed upregulation of 2 different viral mRNA transcripts, p41 and U86 (Fig. 1c). However, none of the viral transcripts were abundant enough to be detected by Northern hybridization. In addition, we could not detect any of the viral proteins by immunoblotting, immunostaining or mass spectrometry. Immunoprecipitation analysis of the cells treated with TSA could barely show expression of only ~55 kDa variant of immediate early protein (IE-2) (Fig. 1d). This isoform of IE-2 has been previously described.^23^ We did not observe any increase in viral DNA replication up to 2–3 days of TSA treatment. On contrary viral DNA was lost upon TSA treatment within first 3 days of activation (Fig. 1e).

HHV-6A and 6B encoded sncRNAs have been described during active viral infection^17,18^ but nothing is known about their transcription during latency. Hence we checked their transcription profile in our cell culture system using Northern blotting. Interestingly, we detected abundant expression of most of the viral sncRNAs upon TSA treatment in U2OS cells (Fig. 1f, S1) whereas these sncRNAs were not detected in solvent control (DMSO) treated samples except for sncRNA-U86, which was detected in low amount even in solvent control treated samples. Thus our results showed a unique stage in viral life cycle that we termed as transactivation where viral genome is activated leading to transcription of several viral sncRNAs and a few other viral RNAs in the absence of detectable viral DNA replication and protein synthesis.

### Various drugs and pathogens have the potential to induce viral transactivation

We showed that RFP expression from viral genome correlated with regaining of viral transcriptome upon TSA treatment. Numerous studies indicate reactivation of ciHHV-6 as a cause in drug induced hypersensitivity syndrome (DIHS) and other drug related complications.^24,25^ We have also previously shown *Chlamydia* infection-mediated reactivation of latent HHV-6A in various cell types.^26^ Hence, we screened a panel of drugs, hormones and pathogens for their ability to transactivate latent HHV-6 using our in vitro U2OS latent HHV-6 cells carrying either a RFP or a GFP reporter. For this purpose, cells were seeded in 6-well cell culture plates and were treated with multiple different concentrations of the drugs for 24–48 h. Appropriate solvent controls were used in parallel to see background fluorescence activity. Viral activation as assessed by GFP (Fig. S2A) or RFP expression (Fig. S2B) was studied under fluorescence microscope. Fluorescent cells were counted and were normalized to solvent control treated cells. Drugs such as suberoylanilide hydroxamic acid, escitalopram oxalate and hormones such as oxytocin were found to be efficacious in activating ciHHV-6A (Fig. S2A). Corroborating the fluorescent signal, viral mRNA analysis (Fig. S2C and S2D) revealed concurrent increase in viral IE transcripts as observed with TSA treatment. Detailed information on different drugs and pathogens tested and their ability to transactivate latent HHV-6 is summarized in Table S1. Our results show that several frequently used prescription drugs have the potential to activate HHV-6 transcription.

### Characterization of early stages viral transactivation

We followed high throughput transcriptomics approach to understand viral transcriptome during transactivation. Separate sequencing studies were carried out to evaluate sncRNAs as well as mRNAs. Biological duplicate samples prepared on two different days were processed for each study with appropriate controls. DMSO and TSA treated U2OS cells without having viral genome were used for normalizing the sequencing data for further analysis.

#### Viral mRNA profile upon HHV-6A transactivation

mRNA library was prepared from both solvent (DMSO) control treated as well as TSA treated U2OS cells carrying latent HHV-6A or without having any viral genome. Next generation sequencing revealed minor changes in viral transcription upon TSA treatment (Fig. S3). Interestingly, TSA-mediated viral transactivation in U2OS cells revealed increased transcription of only 3 viral ORF (U77/U79, U90 and U91; Fig. S3C-S3D; Table S2). We detected transcription of U7 ORF (homologous to HCMV UL28) during viral latency, which is suppressed upon transactivation (Fig. S3B). We did not detect U94 transcripts during latency as well as upon viral transactivation.

#### Viral sncRNA transcription upon HHV-6A transactivation

As we detected transcriptional activation of several viral sncRNAs upon TSA treatment (Fig. 1f) in the absence of major alterations in rest of the viral transcripts, we sequenced both viral and human small RNA transcriptome separately using same sets of RNAs that was used for mRNA transcriptome analysis. At least 30 million processed sequencing reads were generated from each sample for further analysis. Sequence analysis of viral small RNA transcriptome revealed transcription of several previously described small non-coding RNAs only upon TSA-mediated viral transcactivation. However, sequencing depth was not enough to detect all of the previously described small viral non-coding RNAs in abundance. sncRNA-U14 was the most abundant in our sequencing results supporting the Northern blot results (Fig. 1f, S1). Interestingly, we detected a new 18 nt long viral sncRNA encoded from the U73 ORF (henceforth called as sncRNA-U73), which was transcribed only upon TSA-mediated viral transactivation. Northern blot analysis confirmed the sequencing results (Figs. 1g, h). Thus our results showed transcriptional regain of function of parts of viral genome upon transactivation, which was not associated with immediate viral DNA replication.

### Impact of viral transactivation on host cell physiology

Loss of viral genome, lack of major increase in viral replication and protein expression suggested negligible impact of viral transactivation on host cell. To further evaluate this, we analyzed human transcriptome in the previously described set up. Viral transactivation in U2OS cells was associated with upregulation and downregulation of several human transcripts (Fig. S4). Altered transcriptome profile of HHV-6A transactivation was compared to known molecular pathways using publically available datasets like gene ontology (GO) and Kyoto Encyclopedia of Genes and Genomes (KEGG) pathways (Table S3), which suggested a complex effect of viral transactivation on host cell.

**Fig. 2:**
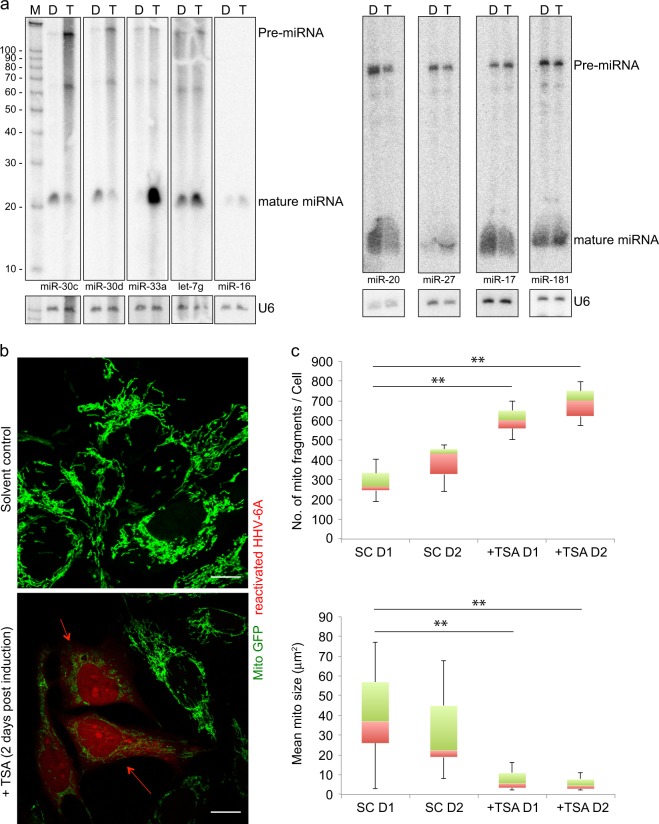
HHV-6 alters host miRNA expression and mitochondrial dynamics. **a** Transcription dynamics of several human miRNAs were studied by Northern hybridization. U2OS cells having latent HHV-6A were used as described in figure 1f. Decade marker (M) was used to verify sizes of identified RNA. Both matured miRNA and their precursor (pre-miRNA) are indicated. Human U6 RNA was used as loading control. All gels derive from the same experiment and they were processed in parallel. **b** HHV-6A transactivation induces mitochondrial fragmentation. U2OS cells having soluble GFP (mito GFP) within mitochondria and carrying mCherry encoding latent HHV-6A was reactivated with TSA. DMSO treated cells served as solvent control. 2 days after treatment, cells were fixed and processed for confocal microscopy. The scale bars represent 10 μm. **c** Mitochondrial size and numbers were quantified using ImageJ. Data represent the mean ± SEM of three independent experiments. *SC* solvent control, *TSA* trichostain A, *D1* Day1, *D2* Day 2

Similar analysis of human miRNA transcriptome revealed substantial changes in human miRNAome upon TSA-mediated viral transactivation (Fig. S5A, S5B). We selected several abundantly transcribed human miRNAs and analyzed them by Northern blotting, which corroborated the sequencing results (Fig. 2a).

### HHV-6A transactivation is characterized by increased mitochondrial fragmentation

Analysis of GO biological processes and KEGG pathway, suggested major changes in mitochondria associated pathways upon HHV-6A transactivation. Careful analysis of virus transactivated cells also revealed major changes in mitochondrial morphology. Hence we developed U2OS cells carrying latent HHV-6A and expressing soluble GFP within mitochondria^27^ for quantitative analysis of microscopy-based mitochondrial morphology. This system allows quantitative analysis of mitochondrial morphology without additional requirement of mitochondrial staining. We transactivated HHV-6A in these cells using TSA that led to expression of HHV-6A BAC-encoded RFP proteins. This allowed us to compare cells with virus transactivation to that without it. Upon HHV-6A transactivation, we observed increased mitochondrial fission in U2OS cells (Fig. 2b) that was associated with increased mitochondrial number but decreased mitochondrial size (Fig. 2c). These studies support the transcriptomics analysis results and hints towards potential deregulation of mitochondrial dynamics upon viral transactivation.

### HHV-6 sncRNA-U14 serves as an ideal biomarker for clinical diagnosis of viral reactivation

Our in vitro studies pointed towards importance of HHV-6A encoded sncRNAs as potential markers for viral reactivation studies. Moreover, non-coding RNAs are comparatively stable than other mRNAs making them efficiently detectable even in formalin-fixed paraffin-embedded (FFPE) samples. Hence we developed a locked nucleic acid (LNA)-based probe against sncRNA-U14 to be utilized in fluorescence-in-situ-hybridization (FISH) and analyzed panels of in vivo FFPE human tissue biopsies. To start with, we tested the efficiency of detection of sncRNA-U14 in FFPE-cells with or without HHV-6A infection. In parallel, we also tested the same cells for a scrambled RNA as a negative control and for human small RNA U6 as a positive control. FISH assay was optimized to fit the three probe criteria (Fig. S4A). Next, we validated sncRNA-U14 FISH in HHV-6 PCR positive liver FFPE tissues (Fig. S4B) whereas PCR negative FFPE-liver biopsies gave no signal for sncRNA-U14. We tested sncRNA-U14 as a marker of active viral infection/reactivation in various types of human tissues. Here, we present a unique clinical case of Drug reaction with eosinophilia and systemic symptoms (DRESS)-associated death where we detected HHV-6A sncRNA-U14 in various post mortem tissue biopsies in the absence of detectable amounts of viral late proteins.

**Fig. 3:**
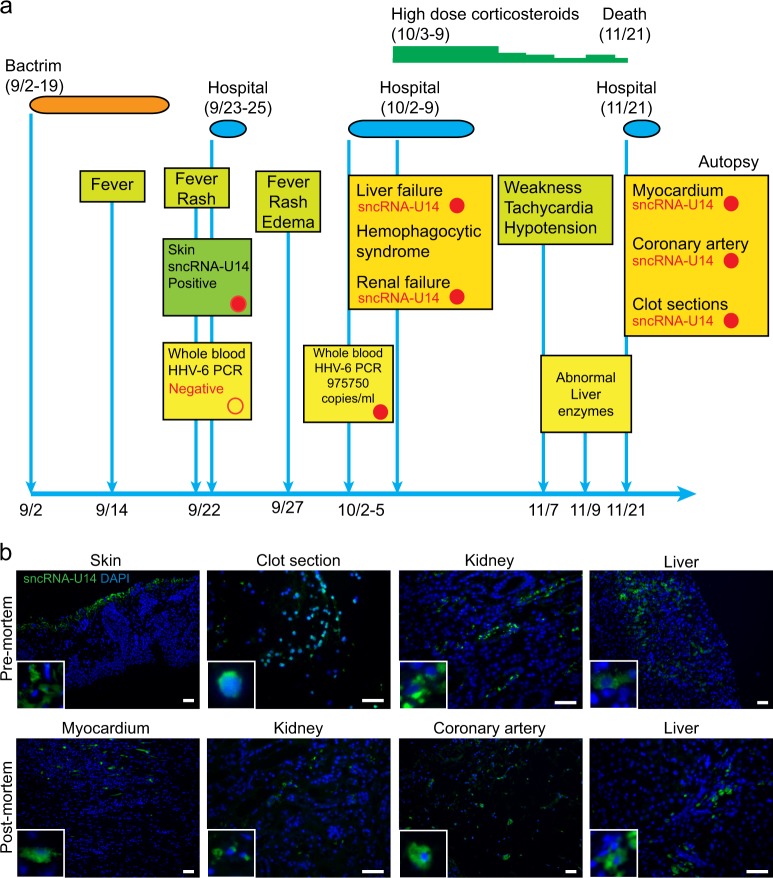
HHV-6 as a potential cause of DRESS-mediated death. **a** Brief summary of all the clinical conditions and different clinical analysis carried out in the DRESS patient is presented in the form of a schematic diagram. Blood and tissue biopsies analyzed for HHV-6 at various stages of the treatment are indicated. HHV-6 positive analyses are indicated with red color filled circles. HHV-6 negative analyses are indicated with white color filled circles. **b** Various types of pre-mortem and post-mortem FFPE tissue biopsies from the DRESS patient were analyzed for sncRNA-U14 by FISH analysis. Blown up images of the positive stained cells are shown within white boxes wherever necessary. Imaging was done on a SP5 confocal microscope. The scale bars represent 200 μm

A 15-year-old girl presented with fever and rash in September 2015 at VCU Health Center, Richmond, USA. She had been treated for acne by an antibacterial, Bactrim for 18 days (Fig. 3a, Table S4). She had no other major medical history prior to this. The treatment had been stopped 3 days before by her dermatologist when she developed a fever. The entire summary of the case is presented in table S4. A blood test was performed, which demonstrated reactive lymphocytosis as confirmed by smear review. At that time HHV-6 and HHV-8 quantitative PCR analysis was negative in whole blood. Serology for CMV (both IgG and IgM) and EBV was negative except for EBV Capsid Ag IgG, which was positive. During the following days, the rash spread with facial edema and the fever rose to 104 degrees with malaise. She was re-admitted to the hospital in October 2015. She had a pruritic generalized morbilliform exanthema with edema and jaundice without lymphadenopathy. Her blood tests demonstrated an acute liver failure with cytolytic and cholestatic hepatitis (AST 2.009 U/L (*N* < 50), ALT 1.386 U/L (*N* < 50), alkaline phosphatases 1.110 U/L (*N* < 120)). There was no eosinophilia but it always noted a mononucleosis syndrome: lymphocytosis with reactive lymphocytes (3.7 10e9/L). Ferritin was elevated at 5075/L (*N* < 150) associated with hypertriglyceridemia. Abdominal ultrasound showed a hepatosplenomegaly. HHV-6 quantitative PCR in whole blood demonstrated a very high level of HHV-6 DNA (975750 copies/mL). EBV, CMV, and parvovirus serologies were negative. EBV capsid Ag IgM was doubtful; hence EBV DNA PCR was carried out, which was also negative. A skin biopsy showed spongiosis with interface dermatitis. Working diagnoses included hemophagocytic lymphohistiocytosis, viral infection, and Bactrim-induced DRESS syndrome. She was started on high dose corticosteroids (80 mg/day of prednisone). Liver, kidney and bone marrow biopsies were performed in October. The bone marrow biopsy showed small amount of hemophagocytosis. Tests for studying NK cell cytotoxic function did not demonstrate any alteration. No eosinophil was observed in liver and renal biopsies but these biopsies were performed after corticosteroid initiation. IL2 receptor alpha levels were found to be high. Afterwards she was released from hospital. There was a rapid clinical and biological improvement with corticosteroids. The liver tests returned close to normal values. The corticosteroids were progressively tapered (details in Table S4). Concomitantly to the weaning off steroids to 10 mg per day she became more and more tired, had hypotension and tachycardia. A slight increase of liver enzymes and inflammatory marker (C reactive protein) was noted. Her condition was getting worse hence she was admitted to the emergency department. 1 h after her admission, she suddenly had cardiac dysrhythmia leading to cardio-respiratory arrest. Subsequently an autopsy was performed. Cause of death was reported as eosinophilic myocarditis. Final clinical diagnosis was drawn as Bactrim-induced DRESS complicated by multi-organ failure, including liver failure, hemophagocytic syndrome, and myocarditis. Diagnosis of DRESS syndrome was established as a definite case by applying RegiSCAR scoring system showing a score of 6 (Table S5). Diagnosis as an atypical DIHS (Drug Induced Hypersensitivity Syndrome) was also established according to the Japanese Consensus Group scoring system.

As HHV-6 was the only pathogen that was detected with very high viral load during the course of the disease, DNA qPCR and FISH analysis was carried out to look for HHV-6 infection in blood as well as pre-mortem and post-mortem tissue samples. Only 1065 copies of HHV-6A DNA per million blood cells were detected in frozen blood derived DNA, which was collected at the time of death. HHV-6B was not detected in the blood. No viral DNA was detected in post-mortem kidney, skin biopsies except for post-mortem liver biopsy where log20 fold HHV-6A DNA was detected in the absence of HHV-6B. In contrast, HHV-6 sncRNA-U14 was detected in liver, myocardium, kidney, and coronary artery biopsies by FISH analysis (Fig. 3b). We then traced back the biopsy samples collected during the mid-stage (second hospitalization) of the disease (October, 2015). FFPE-biopsies taken from skin at the very beginning of the disease, clot, kidney and liver were positive for HHV-6 sncRNA-U14. These results point towards potential use of HHV-6 sncRNA-U14 as a biomarker for early detection of viral activation in clinical materials.

## Discussion

HHV-6 latency and activation is a heterogeneous process. At any point of time only a fraction of infected cells carry the integrated or reactivated viral genome.^28,29^ Hence it becomes important to use a model system that can be utilized to obtain uniform cell populations upon viral activation carrying activated viral genome. We developed a BAC-derived latent HHV-6A carrying U2OS cell line for studying viral reactivation. Such a system is recently described by another laboratory independently.^21^ Viral reactivation in U2OS cells is comparatively easier as these cells utilize ALT mechanism for telomere maintenance and hence frequently form telomeric circles under normal growth conditions.^30^ The process of telomeric circle formation can be efficiently induced in these cells, which also facilitates faster viral genome excision through host cell telomeric circle formation machinery.^31^ Using these cells we studied if viral reactivation can be quantified through reporter GFP/RFP signal that is encoded from the viral genome. Upon treatment of a HDAC inhibitor, TSA, which induces histone acetylation, we observed appearance of fluorescence signal in up to 50% of the cells. We also detected transcription of several immediate early viral ORFs at the same time when GFP protein was expressed. Even though GFP/RFP expression and viral transcription are possibly two independent processes and are regulated by different promoters, the co-appearance of both the products encoded by the viral genome makes GFP/RFP as an ideal reporter for studying viral reactivation in these cells. We used these cells for a small-scale drug-screening assay and detected several potential drugs that can reactivate HHV-6A. Drug-induced viral reactivation was further validated by detection of several viral IE transcripts. Various studies have advocated reactivation of HHV-6 in patients upon treatment with particular drugs as a major cause of drug induced hypersensitivity syndrome/drug rash with eosinophilia and systemic symptoms (DIHS/DRESS).^25,32^ Our recent study has shown the possibility of progesterone induced reactivation of iciHHV-6 in a pregnant woman causing complications in the new born.^33^ This highlights the necessity for careful scrutiny of prescription drugs prior to administration to iciHHV-6 individuals. We envisage that a drug-screening platform like ours would play a significant role in prophylaxis by aiding in determining an ideal drug treatment for iciHHV-6 individuals. This may be also proposed to patients with previous severe DIHS/DRESS with HHV-6 reactivation. For instance patients with epilepsy who suffered from anticonvulsant-induced DIHS/DRESS are very anxious to develop a new severe reaction after introducing a new anticonvulsant treatment. A drug-screening platform is warranted for these patients.

We selected a homogeneous clonal population of cells to obtain equal effect of reactivation stress in every cell. However, we observed viral transactivation in only 30–50% cells as rest of the cells did not express any fluorescence signal. Furthermore, viral DNA copy number per cell was also reduced by ~70% after TSA treatment accompanied by increased cell death in those cells, which expressed fluorescence reporter proteins. We have recently shown that HHV-6A integration is followed by partial or complete excision of integrated viral genome.^28^ Similar loss of viral genome in selected tumor tissues was also reported in iciHHV-6 individuals.^34^ Differential amount of decrease in viral copy number after initial reactivation stimuli are documented by several studies.^22,26^ Kondo et al have documented presence of viral transcripts only in a small proportion of cells.^8^ We suggest that the excised viral genome is possibly detected and eliminated by host cell innate DNA/RNA-sensing machinery in majority of the cells.^35,36^

Our studies show lack of complete and productive viral reactivation in U2OS cells that are possibly non-permissive for HHV-6 replication. However we show that viral genome excision in these cells and gain of partial function by viral genome (transactivation) might be clinically significant. Through high throughput transcriptomics, we showed here that transactivation of HHV-6 is capable of bringing in major changes in host cell transcriptome. Interestingly, changes in human transcriptome matched to various previously documented alterations in metabolic pathways. Furthermore, we observed increased mitochondrial fission in HHV-6A transactivated cell, which provides possible explanation for changes in various mitochondria-associated metabolic pathways. Various human miRNAs have been implicated in intraorganellar mechanisms where they perform the job of fine-tuning of functions required to fulfil the metabolic demands of an organ or cell type. Our deep sequencing approach revealed major changes in expression pattern of a large panel of human miRNAs that hints to a broad range of effect on mitochondria and associated metabolic functions upon viral activation. We detected significant changes in the expression pattern of miR-33 and miR-24-2-5p upon viral transactivation. miR-33 plays crucial role in cholesterol metabolism^37^ whereas miR-24-2-5p plays key role in fatty acid metabolism.^38^ High-fat diet (HFD) plays a central role in the initiation of mitochondrial dysfunction that significantly contributes to skeletal muscle metabolic disorders in obesity. miR-375 has been involved in the regulation of skeletal muscle metabolism. Systemic injection of miR-375 inhibitor to HFD-fed mice prevented the development of detrimental effects of HFD on intestinal transit and enteric neurons, providing direct evidence for the role of miR-375 on modulating ER stress and mitochondrial function.^39^ Interestingly we observed log3.4 fold upregulation in miR-375 upon viral activation indicating potential alterations in fatty acid metabolism. Similarly, miR-195, which has been reported to induce mitochondrial degeneration and ATP reduction in cardiomyocytes^40^ was altered upon viral activation. Several studies have reported the regulation of OXPHOS by miRNAs, particularly miR-210 downregulating Complex III in mitochondria; miR-181c, miR-210 and miR-338 downregulating Complex IV; miR-141 downregulating Complex V; miR-338 regulating COX IV and miR-210 regulating COX 10 in the mitochondrial electron transport chain.^41,42^ Our results show significant changes in all these miRNAs suggesting an effective modulation of electron transport chain upon viral activation. Human miRNAs like miR-34c, miR-499 and miR-140 are directly involved in mitochondrial morphology and structural dynamics. A significant modulation of the expression of these miRNAs by viral transactivation suggested a potentially important effect on mitochondrial function upon viral transactivation.

We observed an intermediate stage in viral reactivation that is characterized by increased transcription from a very few selected viral loci including that from the U90 (IE-1)-U91(IE-A) locus. Active transcription from this locus upon viral reactivation is well documented.^8,43^ Our data show initiation of transcription from U77/U79 locus that codes for a DNA helicase/primase complex. This points towards a possible need for the virus reactivation machinery to modulate both host and/or viral DNA for further viral DNA replication. Interestingly, Nukui et al observed transcription of sncRNA-U77 from this locus during active viral infection.^17^ We for the first time detected transcription from the U3-U7 cluster during viral latency, which was suppressed upon viral transactivation suggesting potential role of these ORFs in viral latency. Furthermore our hypothesis is supported by the finding of Kondo et al who did not observe any defect in viral replication upon deletion of U3-U7 cluster.^44^ We did not observe any differences in U94 transcription during viral transactivation suggesting no potential role for this gene product in viral latency and early reactivation.

We detected transcription of several viral sncRNAs (sncRNA-U2, sncRNA-U14, sncRNA-U54) upon viral transactivation indicating potential importance of these small RNAs in viral life cycle. Interestingly, we observed transcription of miR-U86 even during viral latency, which was further upregulated during transactivation. This partially corroborates finding by Nukui et al suggesting inhibitory role of miR-U86 that suppresses expression of HHV-6 U86^17^ and supports viral latency over lytic infection. However, increased transcription of miR-U86 upon viral transactivation fails to explain this effect. We for the first time also show that many of the small non-coding RNAs described separately for HHV-6A and HHV-6B^17,18^ are shared by HHV-6A during viral transactivation. Our study has identified a new sncRNA (sncRNA-U73) that is transcribed upon viral transactivation from a coding region of origin binding protein.^45–47^ As others did not detect this sncRNA during active viral infection, we propose that this might have a transient role in initial stages of viral activation.

During the course of our study, we came across a unique case of DRESS-associated death, where high copies of HHV-6A DNA was detected in whole blood in the absence of EBV and CMV infection. The high viral DNA load was ignored due to lack of knowledge. We analyzed FFPE-biopsies of the patient collected before and after death for HHV-6 sncRNA-U14 and found it to express in all the types of biopsy materials suggesting active infection or reactivation of HHV-6A. Interestingly, even if the viral load was very low at the time of death and could only be detected in liver biopsy, all biopsy samples showed positive signal for sncRNA-U14. This indicates potential effectiveness of sncRNA-U14 as a viral biomarker for detection of active viral infection in the body. It is of interest to highlight that sncRNA-U14 was demonstrated in the skin sample at the very beginning of the DRESS concomitantly to the mononucleosis syndrome whereas HHV-6 PCR was negative at that time in whole blood. It demonstrates the high value of the detection of sncRNA-U14 to make the diagnosis of HHV-6 reactivation. Altered levels of Calcium ion, glucose, lactate and triglycerides hinted towards abnormalities in mitochondrial metabolism, which might be due to HHV-6 infection. Hematology counts for WBC, RBC, CRP, monocytes and eosinophil throughout the course of the disease pointed towards continued infection in the body. Hence it is plausible that HHV-6 infection could have led to the DRESS-associated complications and subsequent death.

Our study hints to a unique stage in viral life cycle, which is not yet being understood completely. PCR-based DNA studies would not identify this stage in viral life cycle as a clinically important stage in virus activation. However, molecular impact of this stage of HHV-6 on host cell should not be ignored from clinical point of view. Moreover, we show several small non-coding RNAs being transcribed upon viral transactivation making them suitable candidate to be used as biomarkers for HHV-6 studies.

## Material and methods

### Cell culture

U2OS cells were purchased from ATCC and were cultured in McCoy5A medium supplemented with 10% (v/v) FBS and 200 units/ml penicillin-streptomycin (P-S). The cell line was maintained at 37 °C with 5% CO_2_. HHV-6A BAC clone encoding GFP or RFP was a kind gift from Dr. Yasuko Mori and Dr. Eain Murphy. U2OS cells carrying stable GFP expression within mitochondria were developed as mentioned before.^27^

### Case report

Written informed consent was obtained from the patient’s family for reporting the case report including any picture of the patient.

### Drug screening

Cells were seeded in 6 well plates at 60–75% confluency and allowed to adhere overnight. Next, cells were washed with 1X PBS and starved in appropriate medium without FBS and supplemented with 200 units/ml penicillin and streptomycin for 16–18 h followed by addition of the drug in suitable concentration in media without pH indicator supplemented with 1% (v/v) FBS and 200 units/ml P-S.

### Quantitative real time PCR

Quantitative PCR (qPCR) was performed using PerfeCTa qPCR SuperMix (Quanta Biosciences) on a StepOnePlus real time PCR platform (Applied Biosciences) using manufacturer’s protocol and SYBR Green chemistry. Amplified data was analyzed using StepOne Software v2.1. HHV-6 genome equivalents per cells was calculated by determining host cell number by amplification of the PI15 gene as described before.^23^ The list of primers used for amplifying mRNA transcripts are summarized in supplementary Table S6.

### Flow cytometry

For single cell sorting, cells were trypsinized and suspended in 1X PBS. Cells were sorted based on forward and side scatter characteristics (FSC/SSC) on BD FACSAria II flow cytometer (BD Biosciences). Healthy cells were gated and sorted in single cell population in 96 well plates. The cells were monitored and transferred to a 6-well plate and further maintained as described earlier.

### Immunoprecipitation of IE2

U2OS cells carrying latent HHV-6A treated with solvent control or TSA were lysed in RIPA buffer (50 mM Tris-HCl pH 7.5, 150 mM NaCl, 1% Triton X-100, 1% NP-40, 0.1% SDS, 10% Glycerol and protease inhibitor cocktail (Roche)). Protein lysates were incubated with control Flag antibody (F1804 M2, Sigma) and IE-2 specific antibody (P6H8, NIH AIDS reagent program) for overnight at 4 °C. Subsequently, the lysate antibody mixture is incubated with prewashed Protein-A Agarose beads (Sigma) for 4 h at 4 °C. The beads were then washed at least 5 times with wash buffer containing 50 mM HEPES pH 7.5, 250 mM NaCl and Protease inhibitor cocktail (Roche). Finally, the beads were incubated in 2X Laemmli buffer at 95 °C for 5 min followed by separation of proteins by 12% SDS-PAGE and immunoblotting on a PVDF membrane (GE Healthcare). The membrane was blocked in 10% milk followed by incubation in respective primary and secondary antibodies. After subsequent washes in TBST, the PVDF membrane was developed using ECL solutions (Amersham).

### Nothern hybridization

Northern hybridization for small RNAs were carried out as described before.^27^ Probes for detection of human miRNAs were developed based on the sequences from miRBase.

### Immunoblotting

Immunoblotting was carried out as described before.^48^

### Fluorescence in situ hybridization (FISH) for sncRNA-U14

A double FAM tagged (one at each end)-LNA probe was designed against HHV-6A sncRNA-U14 and was synthesized at Exiqon, Denamrk. For FISH staining, FFPE tissue sections were deparaffinized with xylene, treated with DNAse and Proteinase-K and then hybridized with the LNA probe for 1 h. Slides were washed and counterstained with DAPI and were mounted for imaging analysis. All the image analysis were carried out using a Leica epifluorescence and SP5 confocal microscope. Images were processed using FIJI software. Human small RNA U6 probe (positive control) and a scrambled RNA probe (negative control) was purchased from Exiqon, Denmark.

### Mitochondrial size and number analysis

U2OS cells expressing soluble GFP within mitochondria were developed using previously described protocols from our laboratory.^27^ Software and modified algorithm for mitochondrial size and number measurement are also previously described by us.^27^

### mRNA-sequencing

#### NGS library preparation

The amount of total RNA was quantified using the Qubit 2.0 Fluorometric Quantitation system (Life Technologies) and the RNA integrity number (RIN) was determined using the Experion Automated Electrophoresis System (Bio-Rad). RNA-seq libraries were prepared with the TruSeq Stranded mRNA LT sample preparation kit (Illumina) using both, Sciclone and Zephyr liquid handling robotics (PerkinElmer). Library concentrations were quantified with the Qubit 2.0 Fluorometric Quantitation system (Life Technologies) and the size distribution was assessed using the Experion Automated Electrophoresis System (Bio-Rad). For sequencing, samples were diluted and pooled into NGS libraries in equimolar amounts.

#### Sequencing and raw data processing

Expression profiling libraries were sequenced on Illumina HiSeq 3000/4000 instruments in 50-base-pair-single-end mode and base calls provided by the Illumina Real-Time Analysis (RTA) software were subsequently converted into BAM format (Illumina2bam) before de-multiplexing (BamIndexDecoder) into individual, sample-specific BAM files via Illumina2bam tools (1.17.3 https://github.com/wtsi-npg/illumina2bam).

#### Transcriptome analysis

Transcriptome analysis was performed with the Tuxedo suite. For each sample, NGS reads passing vendor quality filtering were aligned to a combination of the hg38 reference genome assembly provided by the UCSC Genome Browser based on Genome Reference Consortium GRCh38 and the Human betaherpesvirus 6 A (NCBI RefSeq NC_001664) with the TopHat2 (v2.1.1, http://genomebiology.com/2013/14/4/R36/abstract), a splice junction mapper utilizing the Bowtie2 short read aligner (v2.2.9 http://www.nature.com/nmeth/journal/v9/n4/full/nmeth.1923.html). Thereby, “basic” Ensembl transcript annotation from version e87 (December 2016) and the Human betaherpesvirus 6 A annotation parsed from the RefSeq entry served as reference transcriptome. Cufflinks (v2.1.1, http://www.nature.com/nbt/journal/v31/n1/full/nbt.2450.html) allowed for transcriptome assembly, customary including novel transcript structures, on the basis of the reference transcriptome and spliced read alignments, as well as raw transcript quantification. Before differential expression calling with Cuffdiff (included in Cufflinks v2.1.1, http://www.nature.com/nbt/journal/v28/n5/full/nbt.1621.html), transcriptome sets of each sample of each group to be compared were combined via the Cuffmerge algorithm. Finally, the cummeRbund (http://www.bioconductor.org/packages/release/bioc/html/cummeRbund.html) and biomaRt (http://www.bioconductor.org/packages/release/bioc/html/biomaRt.html) Bio-conductor libraries were utilized in custom R scripts to perform quality assessment and further refine analysis results.

### Sequencing of small RNAs

Sequencing libraries for 24 samples were prepared. First the total RNA was checked for signs of degradation on a 2100 Bioanalyzer instrument (Agilent). Second small RNA stranded libraries from total RNA (1uq RNA per library) were prepared using the CleanTag Ligation Kit (TriLink BioTechnologies). The Bioanalyzer was then used to check the libraries for the intended miRNA fragment peak at 141 bp. After confirmation a Pippin Prep instrument (Sage Science) was used to select the wanted fragment size range. Quality and concentrations of the final libraries were measured by PicoGreen on an infinite F200 instrument (Tecan). Libraries were then sequenced on a NextSeq 500 platform (Illumina) using high-output v2 kits with 75 cycles and producing 38Mio single-end reads in mean per sample.

#### Bioinformatics for miRNA

Reads that passed the chastity filter on the NextSeq 500 were subject to de-multiplexing and trimming of TriLink adapter residuals using Illumina’s bcl2fastq v2 software (v2.19.1). To refine the output, read quality was checked with FastQC software (v0.11.5) and reads shorter 10 bases or longer 25 bases or containing “N” as a base call, were discarded. In a first analysis the cleaned reads of each sample were de-replicated using the USEARCH software (v8.1.1681) resulting in a list of unique sequences annotated with their frequency in the cleaned read data. These lists of sequences were then searched for via Blast in the X83413.1 Human betaherpesvirus 6 A, variant A DNA reference sequences download from the NCBI database. As the size of the database is small an evalue threshold of 0.0001 was set to pre-filter possible hits. The second analysis of the cleaned reads consisted in read mapping to the human reference genome (assembly hg38) using the RNA mapping software STAR (v2.5.1) with a minimum of 16 matching bases between mapped read and reference, a maximum of 1 mismatch and switched off slice awareness. Uniquely mapped reads to miRBase annotated mature miRNAs were then counted with HTSeq software (v0.6.0). The raw counts were then normalized and analyzed for differential miRNA expression with the DESeq2 (v1.12.4) software.

### Statistical analysis

All statistical calculations were performed using GraphPad Prism 6.0. Error bars displayed on graphs represent the means ± SD of three or more independent replicates of an experiment. D’Agostino–Pearson normality test was used to determine whether datasets were normally distributed. Statistical significance was calculated using Student’s *t* test or one-way analysis of variance followed by Tukey’s multiple comparisons test. For image analysis, six or more biological replicates per sample-condition were used to generate the representative data. Western blot, qRT-PCR, Northern blot data presented are representative of at least three independent experiments.

## Electronic supplementary material

Supplementary material

## Data Availability

The authors declare that all the data related to this manuscript will be made available upon request under collaborative agreement.

## Electronic supplementary material

**Supplementary information** accompanies the paper on the *npj Genomic Medicine* website (10.1038/s41525-018-0064-5).
