# Supplementary material for: HHV-6 encoded small non-coding RNAs define an intermediate and early stage in viral reactivation
Source: NPJ Genom Med. 2018 Sep 5;3:25. doi: 10.1038/s41525-018-0064-5 (PMC6125432; doi:10.1038/s41525-018-0064-5)
Supplement: Supplementary file 1 — Supplementary material [file 41525_2018_64_MOESM1_ESM.pdf]

HHV-6 encoded small non-coding RNAs define an intermediate and early stage in viral reactivation

Bhupesh K. Prusty[1#], Nitish Gulve[1], Suvagata Roy Chowdhury[1], Michael Schuster[2], Sebastian Strempel[3], Vincent Descamps[4], Thomas Rudel[1]

Supplementary Figure legend:

Figure S1: Expression of viral sncRNAs upon TSA treatment.

Detection of several different HHV-6A encoded small non-coding RNAs by Northern hybridization. U2OS cells carrying latent HHV-6A were treated with 80 ng/ml of TSA (T) or DMSO (D) for 48 h. 10 μg of total RNA were separated on a denaturing Urea gel for Northern hybridization. Decade marker (DM) was used to verify sizes of identified RNA. Transcription of previously described HHV-6 encoded small non-coding RNAs (labeled as sR) and miRNAs was tested using specific DNA probes. U6 was used as loading control. U6 is indicated with a black arrowhead. Previous signal from other probes in U6 blot is indicated with a "?" mark.

Figure S2: Prescription drugs like Suberanilohydroxamic acid (SAHA) and Escitallopram oxalate (EO) induce HHV-6A transactivation in U2OS cells.

a. Fluorescence microscopy shows appearance of GFP signal suggesting HHV-6A transactivation in U2OS cells carrying latent HHV-6A post treatment with drugs such as Suberanilohydroxamic acid (SAHA), Escitalopram

oxalate (EO). Effective dose of both the drugs are indicated. DMSO treatment was used in parallel as solvent control.

b. Similar studies were also done using RFP-encoding latent HHV-6A in U2OS cells and various other drugs. Appropriate solvent control treated cells were used in parallel.

c. Quantitative real time PCR for 5 different viral mRNAs demonstrates differential upregulation in early viral transcripts post treatment with SAHA as shown figure A. Data represent the mean ± SEM of three independent experiments.

d. Quantitative real time PCR for 5 same different viral mRNAs demonstrates differential upregulation in early viral transcripts post treatment with EO as shown figure A. Data represent the mean ± SEM of three independent experiments.

Figure S3: Transcription dynamics of HHV-6A in U2OS cells.

a. Total RNA from TSA-treated U2OS cells carrying latent HHV-6A were sequenced using illumine sequencing. DMSO treated cellular RNA was sequenced in parallel for comparison. In addition, U2OS cells without having HHV-6A were treated similarly with either DMSO or TSA and sequenced for further normalization of sequencing data. Sequencing data from two biological duplicate samples are combined for representation. Transcription pattern from the entire length of ~159 kb of HHV-6A genome is represented.

b. Transcription profile of viral RNAs within the genomic region of 5-20 kb is shown.

c. Transcription profile of viral RNAs within the genomic region of 50-60 kb is shown.
d. Transcription profile of viral RNAs within the genomic region of 130-160 kb is shown.

Figure S4: Expression of human transcriptome changes upon TSA treatment in U2OS cells carrying latent HHV-6A.

Total RNA from TSA-treated U2OS cells carrying latent HHV-6A were sequenced using illumina sequencing. DMSO treated cellular RNA from the same cells was sequenced in parallel for comparison. In addition, U2OS cells without having HHV-6A were treated similarly with either DMSO or TSA and sequenced for further normalization of sequencing data. Sequencing data from two biological duplicate samples are combined for representation. Scatter matrix plots of upregulated and downregulated genes are represented on left panel whereas box plots for the same are presented on right panel. Each parameter is compared with its counterpart in four panels (U2OS + HHV-6A +TSA vs + DMSO upper panel; U2OS - HHV-6A +TSA vs + DMSO second panel; U2OS - HHV-6A +TSA vs U2OS + HHV-6 + TSA third panel; U2OS - HHV-6A +DMSO vs U2OS + HHV-6 + DMSO lower panel).

Figure S5: Transcription dynamics of human miRNAs in U2OS cells.

a. Heat map showing 30 most downregulated human miRNAs. Size fractionated small RNAs from TSA-treated U2OS cells carrying latent HHV-6A were sequenced using illumina sequencing. DMSO treated small RNA was sequenced in parallel for comparison. In addition, U2OS cells without having HHV-6A were treated similarly with either DMSO or TSA and

sequenced for further normalization of sequencing data. Sequencing data from two biological duplicate samples (Set 1 and Set 2) are shown separately.

b. Heat map showing 30 most upregulated human miRNAs in the same sample sets as mentioned above. Color key for the heat map and histograms is shown on the right panel.

Figure S6: Detection of sncRNA-U14 by FISH in various *in vivo* cell types.

a. HHV-6A infected HSB-2 cells were used as positive control for sncRNA-U14 FISH analysis. Uninfected HSB-2 cells were used as negative control. Human small RNA U6 was used as a FISH positive control. At the same time a scrambled small RNA probe was used as a FISH negative control. Imaging was done on a SP5 confocal microscope.

b. HHV-6 qPCR positive and negative FFPE human liver tissue biopsies were used to detect sncRNA-U14 by FISH.

Figure S1
Prusty et al.

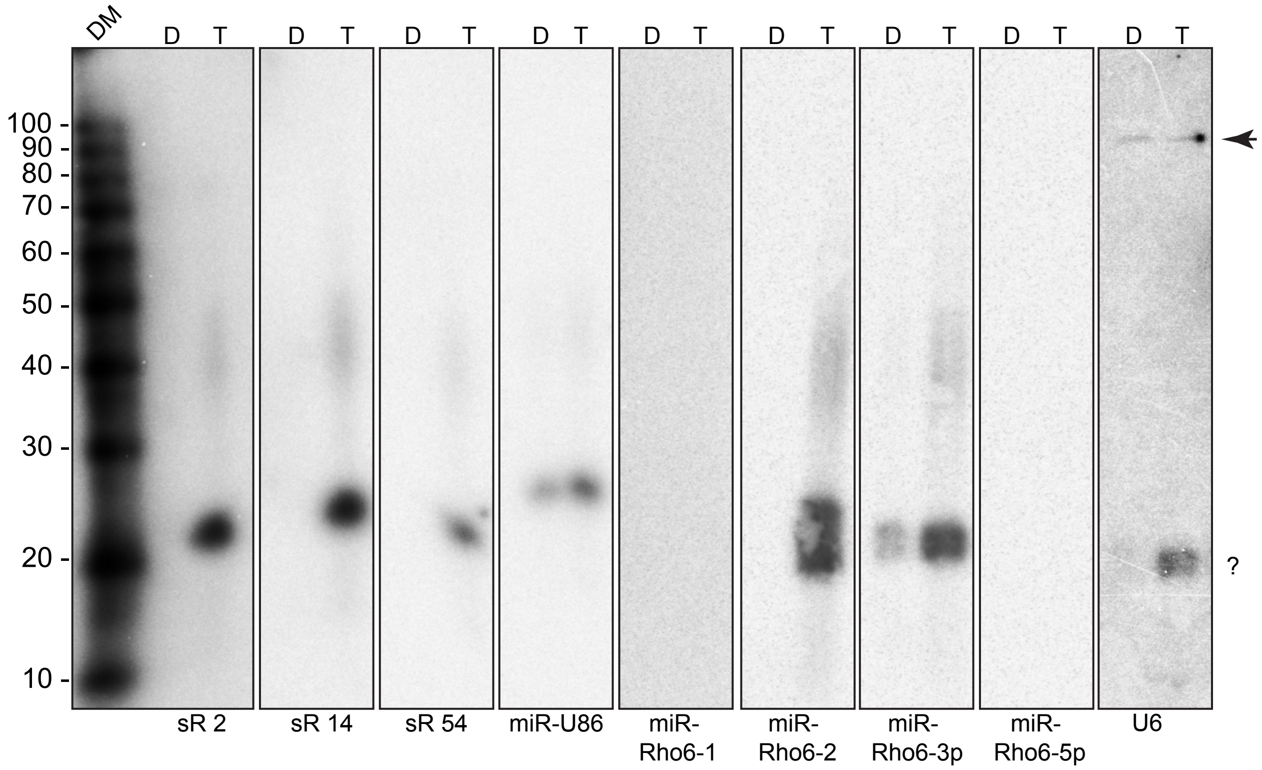

Figure S2
Prusty et al.

a

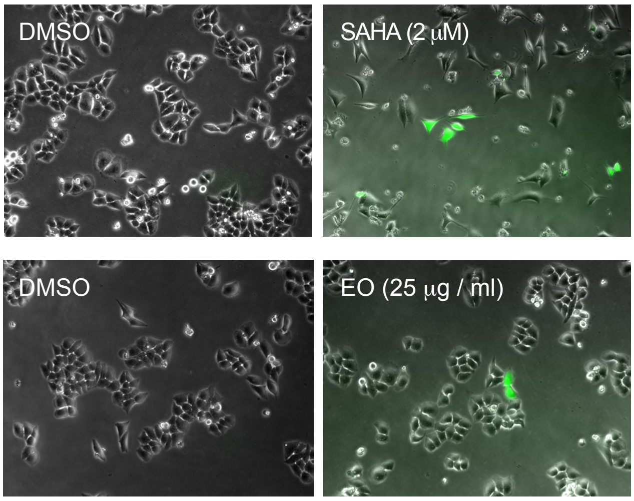

b

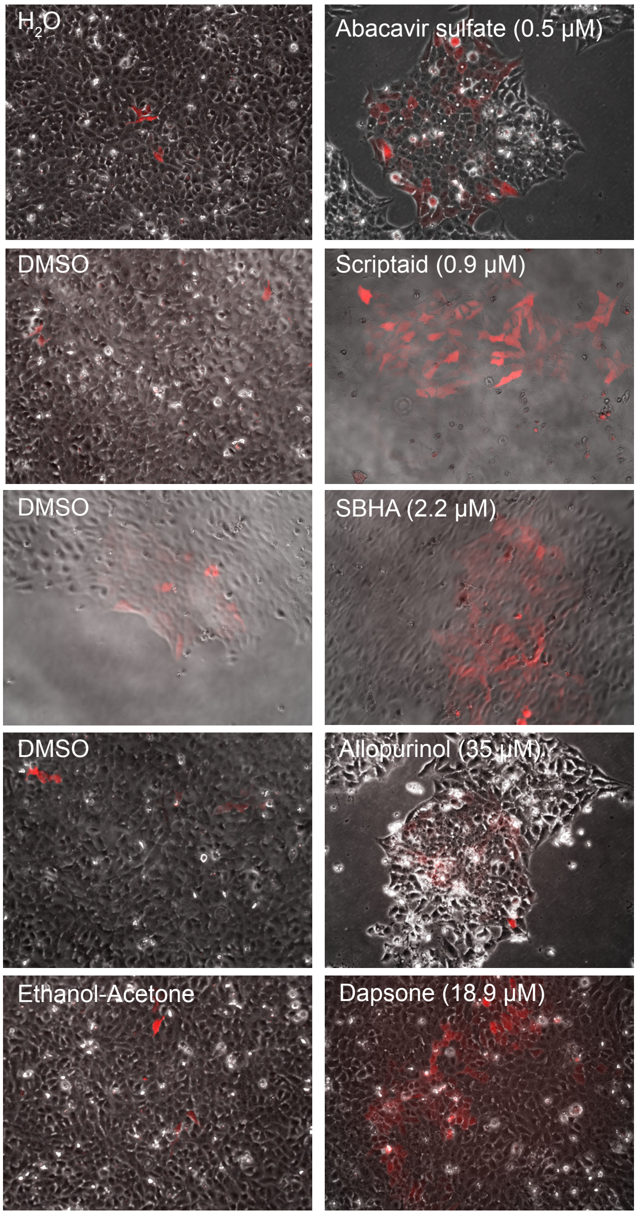

c

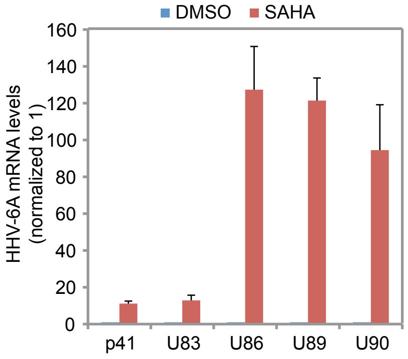

d

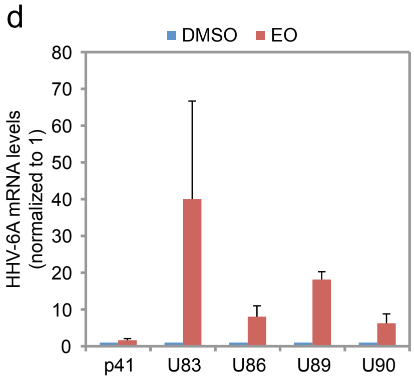

Figure S3
Prusty et al.

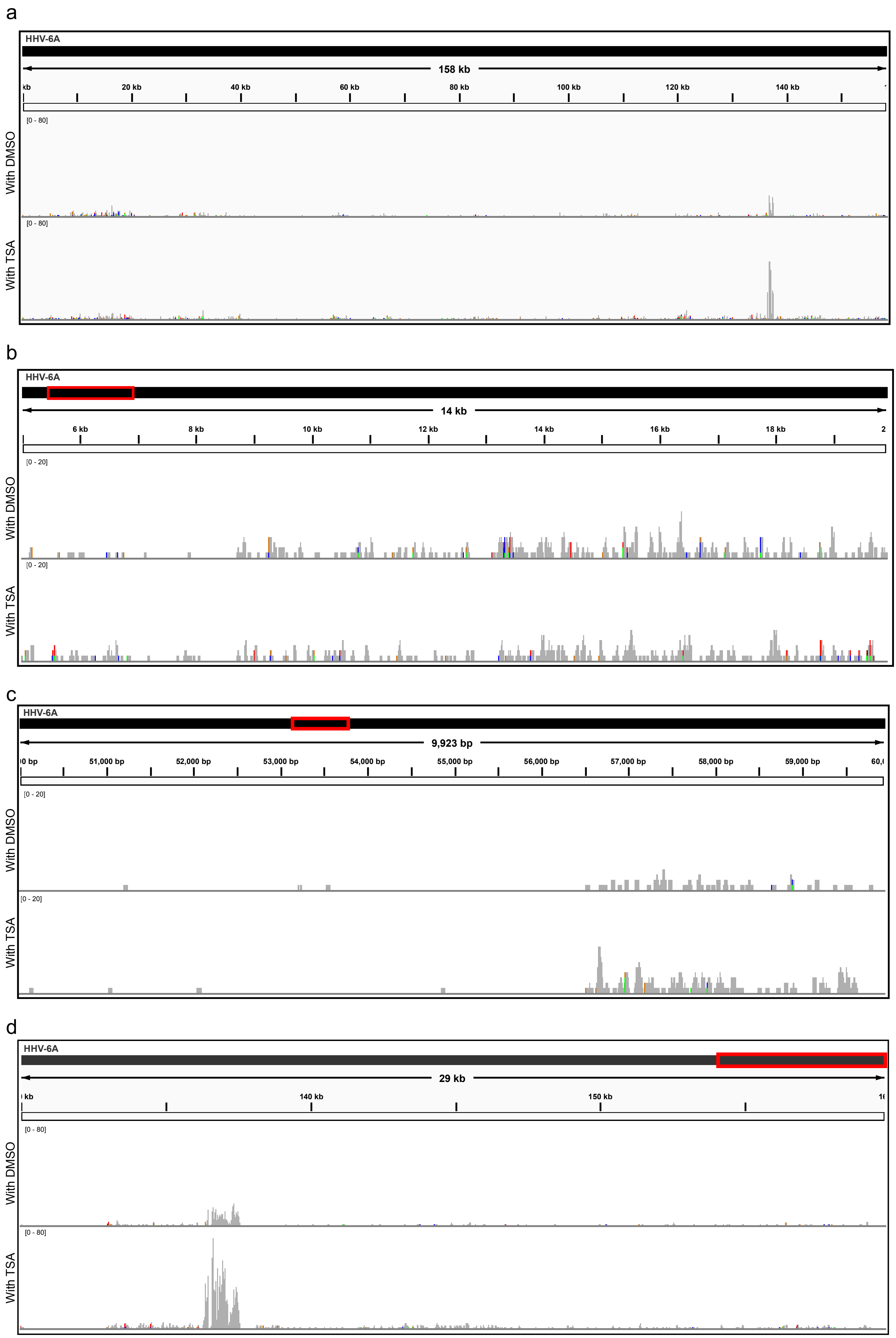

Figure S4
Prusty et al.

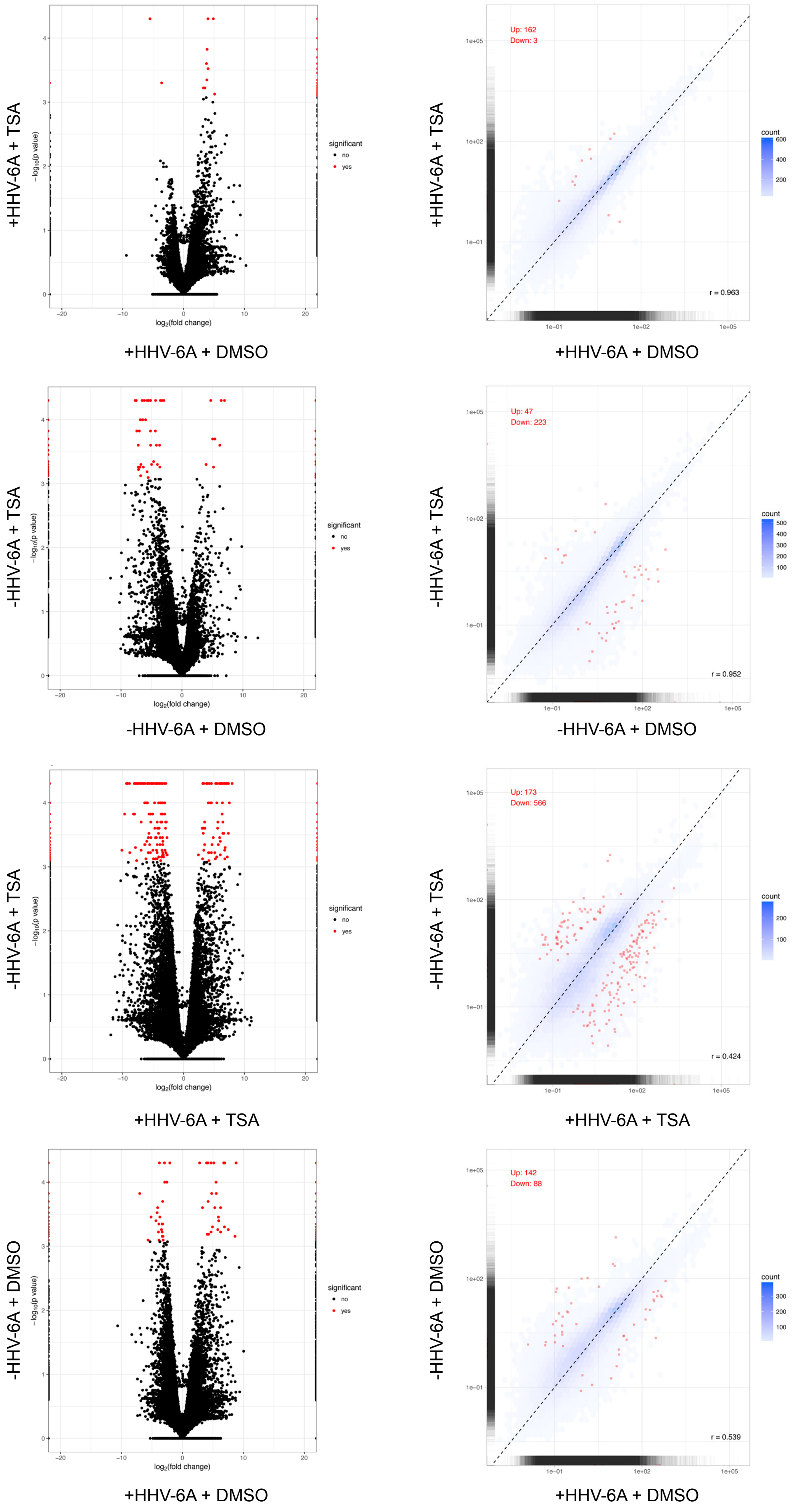

Figure S5
Prusty et al.

a

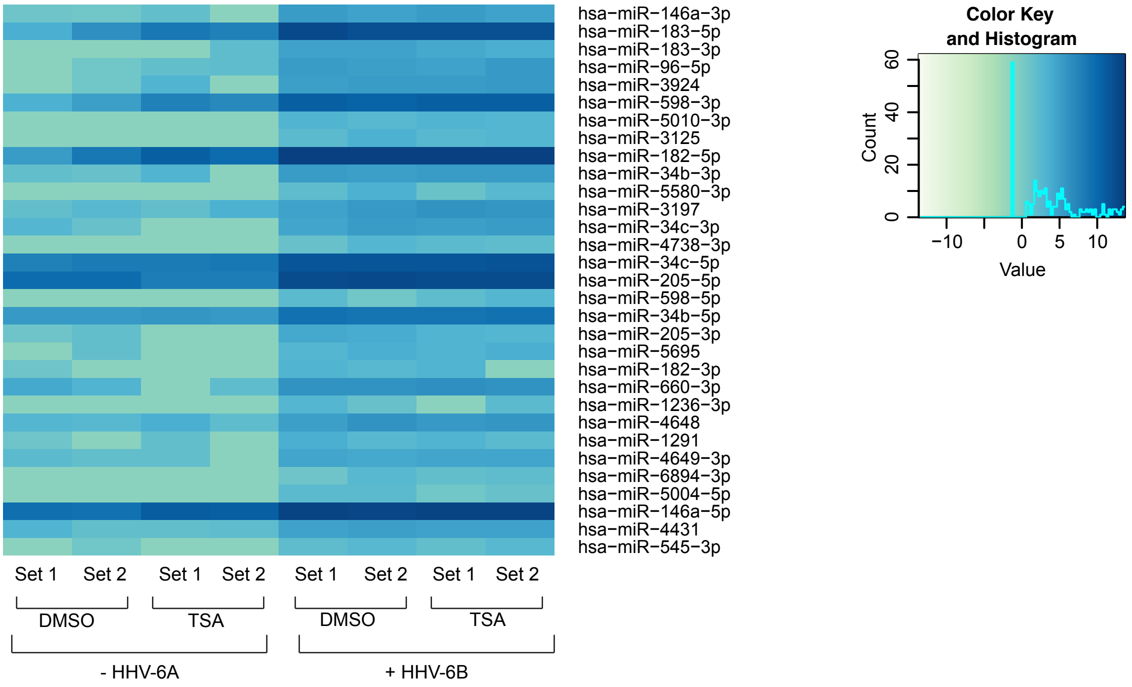

b

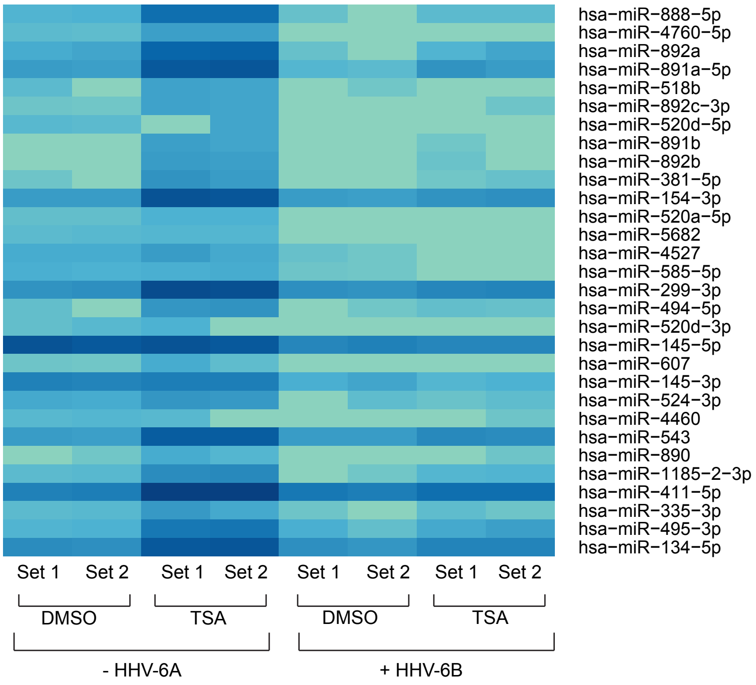

Figure S6
Prusty et al.

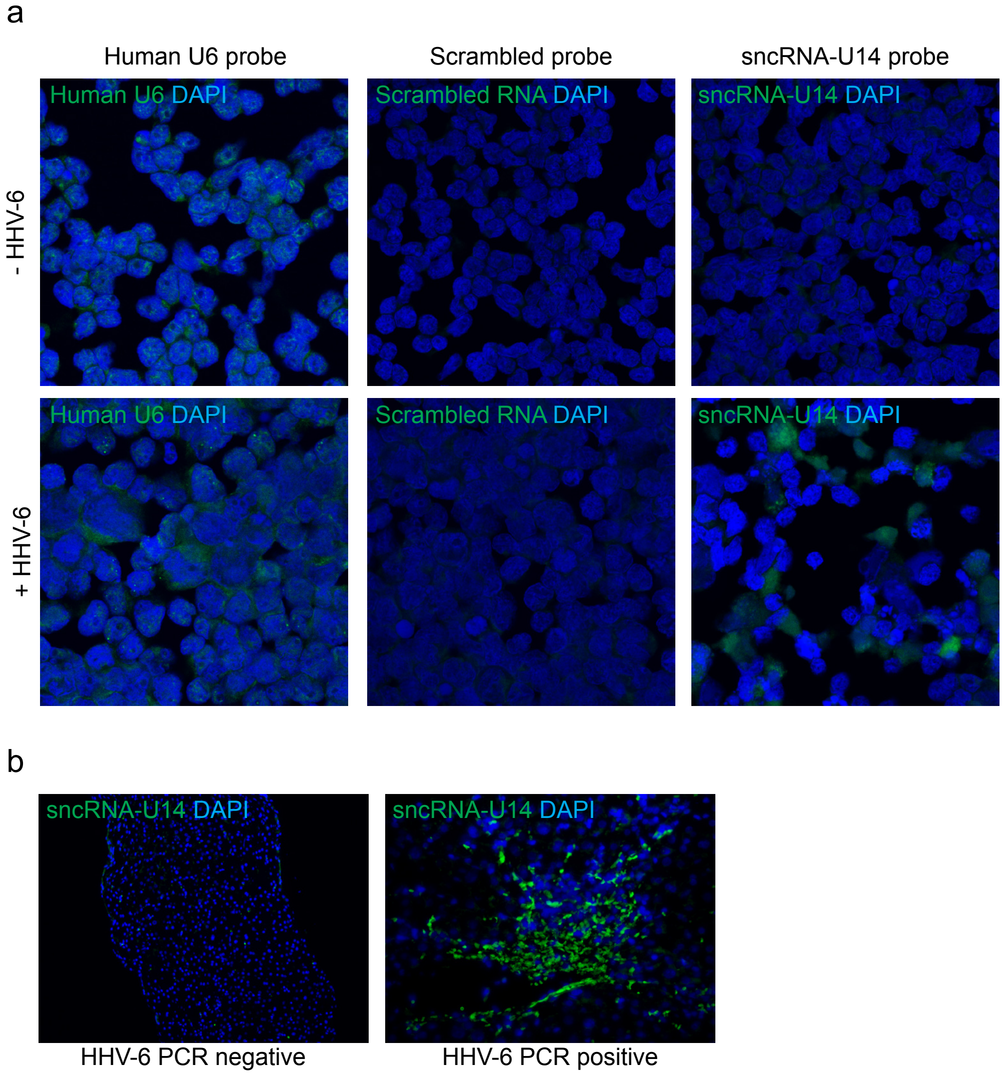

Table S1. List of drugs or pathogens tested for their ability to transactivate HHV-6A using U2OS cells carrying latent HHV-6A. Effective concentrations of the drugs are mentioned within brackets. For pathogenic infections, percentage of activated cells were shown within brackets. hpi, hours post infection.

Drugs with effective concentrations	Viral transactivation
**HDAC Inhibitors**	
Panobinostat	No
SBHA (Suberoyl bis-hydroxamic acid)	Yes (2.2 μM)
TSA (Trichostatin A)	Yes (264 nM)
CI-994 (N-acetyl dinaline)	No
Tubacin	No
SAHA (Suberanilohydroxamic acid)	Yes (100 nM-1 μM)
Scriptaid	Yes (0.9 μM)
Valproic acid	No
**Other Drugs**	
Carbamazepine	Yes (100 μM)
Escitallopram oxalate	Yes (50-100 μM)
Imipramine hydrochloride	Yes (90 μM)
Sulfasalacine	No
Apicidin	No
Allopurinol	Yes (35 μM)
Abacavir sulfate	Yes (0.5 μM)
Minocycline hydrochloride	No
Cetrizine dihydrochloride	No
Docosahexaenoic acid	No
Promethazine	No
Paroxetine hydrochloride hemihydrate	No
Ondansetron hydrochloride dehydrate	No
Setraline hydrochloride	No
**Hormones**	
Progesterone	Yes (2.5-100 ng/ml)
Oxytocin	Yes (10-20 μg/ml)
Hydrocortisone	Yes (0.5-2 μM)
**Antibiotics**	
Gentamycin	No
Ampicillin	No
Amoxicillin	No
Dapsone	Yes (18.9 μM)
**Pathogens (% of activated cells after 72 hpi)**	
Chlamydia trachomatis	Yes (2-5)
Chlamydia pneumoniae	Yes (40)
Chlamydia muridarum	Yes (2-5)
Simkania negevensis	Yes (2-5)

Table S2. Expression of various HHV-6A transcripts in U2OS cells carrying latent HHV-6A.

**HHV-6A transcripts**	**RNA sequence reads detected**	
	**DMSO treated sample**	**TSA treated sample**
U7	101	0.8
U77-U79	0.5	131.7
U90	0.5	119
U91	112	270
U94	0.9	0.4

Table S3. Significantly altered biological processes and pathways during HHV-6 transactivation. Altered human mRNA transcriptome from HHV-6A activated cells were compared with various well established and open access databases including GO database and KEGG pathway. Only statistically significant hits are presented here. Intersting hits possibly linking HHV-6 transactivation to mitochondrial alteration are marked in red.

**GO Biological Process**	**P-Value**
**Analysis of upregulated human transcripts**	
icosanoid metabolic process (GO:0006690)	0,00009955
omega-hydroxylase P450 pathway (GO:0097267)	0,005165
hemidesmosome assembly (GO:0031581)	0,006403
cornification (GO:0070268)	0,045
keratinization (GO:0031424)	0,04154
visual learning (GO:0008542)	0,006403
central nervous system development (GO:0007417)	0,03019
extracellular matrix organization (GO:0030198)	0,008481
leukotriene metabolic process (GO:0006691)	0,01254
regulation of dopamine secretion (GO:0014059)	0,01254
phosphatidylinositol metabolic process (GO:0046488)	0,00405
epidermis development (GO:0008544)	0,01049
epoxygenase P450 pathway (GO:0019373)	0,02734
regulation of G-protein coupled receptor protein signaling pathway (GO:0008277)	0,04652
drug metabolic process (GO:0017144)	0,02983
steroid metabolic process (GO:0008202)	0,04957
**Analysis of downregulated human transcripts**	
establishment of mitotic spindle orientation (GO:0000132)	0,0009662
mitotic sister chromatid segregation (GO:0000070)	0,001966
positive regulation of mitotic metaphase/anaphase transition (GO:0045842)	0,01935
adenylate cyclase-activating dopamine receptor signaling pathway (GO:0007191)	0,01935
regulation of Ras protein signal transduction (GO:0046578)	0,01935
regulation of cell division (GO:0051302)	0,01935
regulation of dopamine uptake involved in synaptic transmission (GO:0051584)	0,01935
cell volume homeostasis (GO:0006884)	0,01935
homocysteine metabolic process (GO:0050667)	0,02318
negative regulation of stress-activated MAPK cascade (GO:0032873)	0,02318
protein insertion into mitochondrial membrane involved in apoptotic signaling pathway (GO:0001844)	0,02318
positive regulation of potassium ion transport (GO:0043268)	0,02699
negative regulation of immune response (GO:0050777)	0,02699
positive regulation of double-strand break repair (GO:2000781)	0,02699
dopamine metabolic process (GO:0042417)	0,02699
positive regulation of synaptic transmission, glutamatergic (GO:0051968)	0,03078
kinetochore assembly (GO:0051382)	0,03078
anoikis (GO:0043276)	0,03456
cellular response to dopamine (GO:1903351)	0,03456
superoxide anion generation (GO:0042554)	0,03833

microtubule depolymerization (GO:0007019)	0,03833
visual learning (GO:0008542)	0,03833
phospholipase C-activating dopamine receptor signaling pathway (GO:0060158)	0,04208
protein O-linked fucosylation (GO:0036066)	0,04208
cell aging (GO:0007569)	0,04208
positive regulation of protein localization to nucleus (GO:1900182)	0,04954
positive regulation of protein export from nucleus (GO:0046827)	0,05325

**KEGG Pathways**	**P-Value**
**Analysis of upregulated human transcripts**	
Complement and coagulation cascades Homo sapiens hsa04610	0,01657
Cell adhesion molecules (CAMs) Homo sapiens hsa04514	0,03167
Histidine metabolism Homo sapiens hsa00340	0,03507
Arachidonic acid metabolism Homo sapiens hsa00590	0,04131
Tyrosine metabolism Homo sapiens hsa00350	0,06926
ECM-receptor interaction Homo sapiens hsa04512	0,08112
Tryptophan metabolism Homo sapiens hsa00380	0,08728
Hematopoietic cell lineage Homo sapiens hsa04640	0,0954
Cocaine addiction Homo sapiens hsa05030	0,1226
Amoebiasis Homo sapiens hsa05146	0,1267
Autoimmune thyroid disease Homo sapiens hsa05320	0,1393
Serotonergic synapse Homo sapiens hsa04726	0,1611
Alcoholism Homo sapiens hsa05034	0,1811
Transcriptional misregulation in cancer Homo sapiens hsa05202	0,1835
Phenylalanine metabolism Homo sapiens hsa00360	0,1905
Axon guidance Homo sapiens hsa04360	0,2075
Dopaminergic synapse Homo sapiens hsa04728	0,2139
Thyroid hormone synthesis Homo sapiens hsa04918	0,2187
One carbon pool by folate Homo sapiens hsa00670	0,2202
Gastric acid secretion Homo sapiens hsa04971	0,2323

Table S4. Summary of course of presentation of the disease and results of various clinical analysis of the DRESS patient. Laboratory test results in red boxes indicate higher than normal range and that in yellow boxes indicate lower than normal range. Written informed consent was obtained from the patient's family for both participation in the study and for the use of patient images in this work.

Date	Clinical symptoms and Test results	Initiated treatment
09.2014 to 09.2015	Presented with Acne	Minocycline
02.09.2015 to 12.09.2015		Bactrim DS
13.09.2015		Bactrim DS + bYaz
14.09.2015-18.09.2015	Low grade fever	Bactrim DS + bYaz
19.09.2015	Fever recedes	Bactrim DS was stopped on advice from the dermatologist.
20.09.2015	Fever	
21.09.2015	Fever	
22.09.2015	Fever with rashes on arms and trunk	Referred to pediatrician
23.09.2015	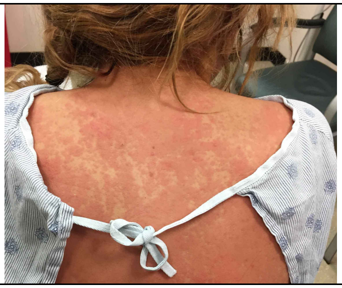 Severe rashes on arms, back and trunk with high fever, nausea and weakness.	Admitted to VCU Medical Center.<br><br>bYaz was stopped.
23.09.2015 to 25.09.2015 **(first hospitalization)**	Suspected DRESS but not confirmed, Liver enzyme levels high at the time of admission but normalized during treatment, facial edema appeared towards the end of the hospital stay.<br><br>HHV-6 PCR in whole blood – negative<br>HHV-8 PCR in whole blood – negative<br>EBV Capsid Ag, IgM – negative	Steroid treatment.<br><br>Discharged from the hospital with prescription of Fluoxetine 20 mg,

<table>
<tr>
<td></td>
<td>CMV Ag, IgG – negative
CMV Ag, IgM – negative
EBV Capsid Ag, IgG – positive
EBV Capsid Ag, IgM – negative
<table>
<tr><th></th><th>23.09.2015</th><th>24.09.2015</th></tr>
<tr><td>ALT</td><td>395 unit(s)/L</td><td>249 unit(s)/L</td></tr>
<tr><td>AST</td><td>137 unit(s)/L</td><td>82 unit(s)/L</td></tr>
<tr><td>Calcium L</td><td>9.0 mg/dL</td><td>7.7 mg/dL</td></tr>
<tr><td>Glucose L</td><td>101 mg/dL</td><td>101 mg/dL</td></tr>
<tr><td>WBC</td><td>9.9 10e9/L</td><td>11.8 10e9/L</td></tr>
<tr><td>RBC</td><td>3.87 10e12/L</td><td>3.78 10e12/L</td></tr>
<tr><td>Hemoglobin</td><td>11.8 g/dL</td><td>11.1 g/dL</td></tr>
<tr><td>HCT</td><td>33.4%</td><td>32.8%</td></tr>
<tr><td>Neutrophil</td><td>5.9 10e9/L</td><td>-</td></tr>
<tr><td>Monocyte</td><td>0.5 10e9/L</td><td>-</td></tr>
<tr><td>Eosinophil</td><td>0.0 10e9/L</td><td>-</td></tr>
</table>
</td>
<td>Lorazepam 1 mg, Zofran 4 mg and triamcinolone 0.1% topical ointment.</td>
</tr>
<tr>
<td>26.09.2015 to 28.09.2018</td>
<td>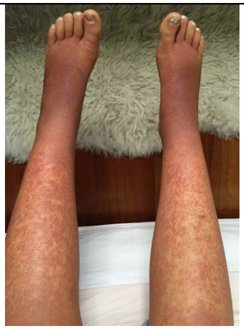 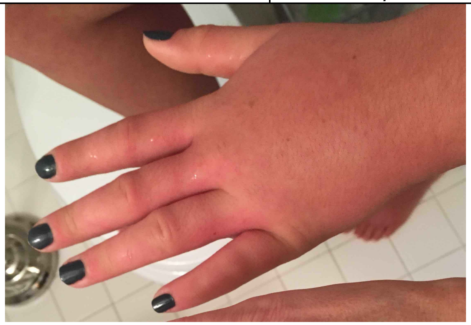
Body swelling increased every day with extreme malaise.</td>
<td></td>
</tr>
<tr>
<td>29.09.2015 to 01.10.2015</td>
<td>Increased body swelling, high fever, vaginal yeast infection. Difficulty in swallowing eating. Patient was diagnosed with thrush and had liquid secretions from both ears.</td>
<td></td>
</tr>
<tr>
<td>02.10.2015 to 09.10.2015 (second hospitalization)</td>
<td>Diagnosed with liver failure along with other systemic involvement.

Suspected Hemophagocytic Lymphohistiocytosis and DRESS but not confirmed.
NK cell function test – negative
Bone marrow biopsy - small amounts of hemophagocytosis.
HHV-6 PCR in whole blood – 975750 copies/ml
EBV Nuclear Ag, IgG – Positive</td>
<td>Started with high dose Steroids</td>
</tr>
</table>

EBV Capsid Ag, IgG – Positive
EBV Capsid Ag, IgM – Equivocal
EBV DNA qPCR – negative
CMV Ag, IgG – negative
CMV Ag, IgM – negative, HAV IgG, Positive, HepB surface Ab – Positive, Parvovirus B19 IgM – Negative.

	02.10.2015	03.10.2015	04.10.2015	05.10.2015	06.10.2015	07.10.2015	08.10.2015	09.10.2015
**ALT**	1386 U/L	1739 U/L	1931 U/L	1670 U/L	1258 U/L	966 U/L	796 U/L	678 U/L
**AST**	2009 U/L	2192 U/L	2391 U/L	1431 U/L	441 U/L	176 U/L	132 U/L	88 U/L
**Calcium L**	7.8 mg/dL	7.0 mg/dL	7.9 mg/dL	7.8 mg/dL	7.5 mg/dL	7.7 mg/dL	7.8 mg/dL	7.4 mg/dL
**CRP**	-	6.4 mg/dL	3.8 mg/dL	-	0.7 mg/dL	0.4 mg/dL	0.3 mg/dL	0.3 mg/dL
**Ferritin**	-	-	5075 ng/mL	2775 ng/mL	922 ng/mL	592 ng/mL	567 ng/mL	594 ng/mL
**Glucose L**	83 mg/dL	74 mg/dL	115 mg/dL	142 mg/dL	179 mg/dL	145 mg/dL	177 mg/dL	163 mg/dL
**Triglycerides**	-	-	217 mg/dL	-	-	392 mg/dL	388 mg/dL	387 mg/dL
**Lactate**	2.7 mmol/L	1.1 mmol/L	-	-	-	-	-	-
**WBC**	8.8 10e9/L	4.5 10e9/L	3.4 10e9/L	5.7 10e9/L	8.6 10e9/L	7.6 10e9/L	7.3 10e9/L	11 10e9/L
**RBC**	3.23 10e12/L	2.90 10e12/L	3.44 10e12/L	3.44 10e12/L	3.1 10e12/L	2.98 10e12/L	3.0 10e12/L	3.40 10e12/L
**Hemoglobin**	9.9 g/dL	9.0 g/dL	8.8 g/dL	10.1 g/dL	9.3 g/dL	8.8 g/dL	8.8 g/dL	9.9 g/dL
**HCT**	28.4%	26.5%	26.6%	30.3%	28.1%	27.4%	27.7%	31.0%
**Neutrophil**	4.3 10e9/L	2.4 10e9/L	-	2.9 10e9/L	4.5 10e9/L	-	-	-
**Monocyte**	0.5 10e9/L	0.1 10e9/L	-	0.2 10e9/L	0.4 10e9/L	-	-	-
**Eosinophil**	0.1 10e9/L	0.0 10e9/L	-	0.0 10e9/L	0.0 10e9/L	-	-	-

	**IL2 Ra**	-	-	7586 U/ml	-	-	4199 U/ml	-	-

09.10.2015

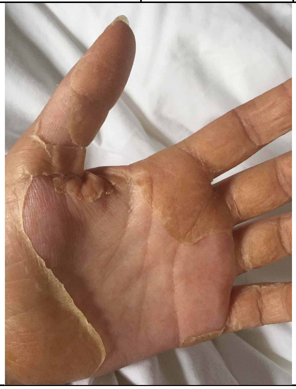

Scaling skins on palm and soles.

Stabilized liver enzymes and inflammatory markers. Released from Hospital. Started with Prednisone 80 mg per day with gradual wean and medication for high blood pressure.

Prednisone 80 mg/day 09.10.2015 to 27.10.2015

10.10.2015 to 07.11.2015

Strong overall improvement

	**(13.10.2015)**	**(20.10.2015)**	**(27.10.2015)**	**L (03.11.2015)**
**ALT**	334 unit(s)/L	123 unit(s)/L	89 unit(s)/L	69 unit(s)/L
**AST**	40 unit(s)/L	27 unit(s)/L	24 unit(s)/L	25 unit(s)/L
**Calcium L**	9.3 mg/dL	9.2 mg/dL	9.7 mg/dL	9.5 mg/dL
**CRP**	< 0.3 mg/dL	< 0.3 mg/dL	< 0.3 mg/dL	< 0.3 mg/dL
**Ferritin**	445 ng/mL	311 ng/mL	164 ng/mL	117 ng/mL
**Gluocse L**	138 mg/dL	85 mg/dL	125 mg/dL	90 mg/dL
**Triglycerides**	406 mg/dL	162 mg/dL	151 mg/dL	148 mg/dL
**WBC**	16.8 10e9/L	17.8 10e9/L	18.5 10e9/L	11.5 10e9/L
**RBC**	3.7 10e12/L	3.9 10e12/L	3.89 10e12/L	3.96 10e12/L
**Hemoglobin**	11.1 g/dL	11.4 g/dL	11.6 g/dL	12.2 g/dL
**HCT**	33.0%	34.7%	34.4%	35.0%
**Neutrophil**	13.5 10e9/L	14.6 10e9/L	15.6 10e9/L	8.9 10e9/L
**Monocyte**	0.8 10e9/L	0.6 10e9/L	0.8 10e9/L	0.7 10e9/L
**Eosinophil**	0.1 10e9/L	0.2 10e9/L	0.1 10e9/L	0.2 10e9/L
**IL2 Ra**	-	754 U/ml	-	-

ketoconazole 2% topical shampoo and betamethasone dipropionate 0.05% topical lotion from 04.11.2015

Prednisone 40 mg/day 27.10.2015 to 30.10.2015

Prednisone 30 mg/day 31.10.2015 to 02.11.2015

08.11.2015 to 14.11.2015

Overall improvement but low blood pressure.

	**09.11.2015**	**19.11.2015**
**ALT**	55 unit(s)/L	54 unit(s)/L

Blood pressure medication discontinued.

	**AST**	23 unit(s)/L	66 unit(s)/L	Prednisone 20 mg from 03.11.2015 to 11.11.205<br><br>Prednisone 15 mg from 12.11.2015 to 14.11.2015
	**Calcium L**	9.2 mg/dL	9.1 mg/dL	
	**CRP**	< 0.3 mg/dL	2.4 mg/dL	
	**Ferritin**	83 ng/mL	-	
	**Glucose L**	75 mg/dL	76 mg/dL	
	**Triglycerides**	140 mg/dL		
	**WBC**	10 10e9/L	11.3 10e9/L	
	**RBC**	3.86 10e12/L	3.83 10e12/L	
	**Hemoglobin**	11.7 g/dL	11.5 g/dL	
	**HCT**	33.9%	33.0%	
	**Neutrophil**	7.0 10e9/L	8.1 10e9/L	
	**Monocyte**	0.5 10e9/L	0.7 10e9/L	
	**Eosinophil**	0.2 10e9/L	-	
	**IL-2 Ra**	386 U/ml	-	
15.11.2015 to 18.11.2015	Increased tiredness			Prednisone<br>10 mg from 15.11.2015 to 19.11.2015
19.11.2015	Got faint, vomiting			Multivitamin<br><br>Prednisone increased to 20 mg on 20.11.2015
		**19.11.2015**		
	**ALT**	54 unit(s)/L		
	**AST**	66 unit(s)/L		
	**Calcium L**	9.1 mg/dL		
	**CRP**	2.4 mg/dL		
	**Eosinophil**	0.2 10e9/L		
21.11.2015<br>**(Third hospitalization)**	Frequent vomiting in the morning. Overall condition declined so back to emergency room. Blood drawn immediately after death and frozen for future analysis.			Admitted to Hospital at 2pm.<br><br>Passed away after seizure like motion and heart failure. Later confirmed as Eosinophilic
		**21.11.2015**		
	**ALT**	150 unit(s)/L		
	**AST**	129 unit(s)/L		
	**Calcium L**	8.1 mg/dL		
	**CRP**	2.4 mg/dL		
	**Cortisol L**	28.6 ug/dL		
	**Ferritin**	680 ng/mL		

	**Glucose L**	170 mg/dL	Myocarditis upon autopsy.
	**Lactate**	3.4 mmol/L	
	**Triglycerides**	190 mg/dL	
	**WBC**	20.4 10e9/L	
	**RBC**	3.40 10e12/L	
	**Hemoglobin**	10.7 g/dL	
	**HCT**	30.1%	
	**Neutrophil**	16.3 10e9/L	
	**Monocyte**	014 10e9/L	
	**Eosinophil**	0.4 10e9/L	
2017	PCR for HHV-6 using FFPE-biopsies and frozen blood. FISH analysis for viral sncRNA-U14 using FFPE-biopsies.<br><br>**HHV-6A DNA in blood DNA – 1065 copies/ 10e6 cells, HHV-6B- negative**<br>**HHV-6A DNA in Liver – 20123233 copies/ 10e6 cells, HHV-6B- negative**<br>HHV-6A DNA in Kidney – below detectable range<br>HHV-6A DNA in Liver – below detectable range		

* Total Prednisone wean from 80 mg - 10 mg occurred in 21 days. Prednisone wean from 20 mg to 10 mg was nine days.

Table S5. RegiSCAR Diagnostic Criteria for diagnosis of DRESS syndrome in the studied case.

Criteria	Score			Minimum	Maximum
**Fever ≥ 38.5**	No (−1)	**Yes (0)**		(−1)	**(0)**
**Enlarged lymph nodes (minimum of 2 sites, at least 1 cm)**	**No (0)**	Yes (1)		**(0)**	(1)
**Eosinophils greater than the normal range**		0.7-1.499 × 10^9 L^{-1} (1)	>1.5 × 10^9 L^{-1} (2)	**(0)**	(2)
**If leucocytes <4 × 10^9 L^{-1}**		10-19.9% Eosinophils (1)	≥20% Eosinophils (2)		
**Atypical lymphocytes**	No (0)	**Yes (1)**		(0)	**(1)**
**Skin rash**				(−2)	**(2)**
**Body surface area affected (%)**	Unknown (0)	**>50% affected (1)**			
**Rash suggestive of DRESS**	No (−1)	Unknown (0)	**Yes (1)**		
**Biopsy suggestive of DRESS**	No (−1)	Unknown (0)	**Yes (0)**		
**Organ involvement (after excluding other causes): liver, kidney, lung, muscle/heart, pancreas, other organs**	No (0)	Unknown (0)	Yes (1) for each organ, up to two organs	(0)	**(2)**
**Resolution of symptoms ≥15 days**	No (−1)	Unknown (−1)	**Yes (0)**	(−1)	**(0)**

**Other potential causes**					
**Antinuclear antibody**		If none positive and ≥3 negatives (1)		(0)	**(1)**
**Blood culture**					
**Serology for HAV/HBV/HCV**					
**Chlamydia and mycoplasma**					
			Final Score	Minimum [−4]	Maximum [9]
					**Final score: 6**<br><2 = no case<br>2-3 = possible case<br>4-5 = probable case<br>>5 = definite case for DRESS syndrome

Table S6. List of oligo used for qPCR and RT-PCR together with their sequence details.

Primers	Sequence (5'->3')
**Primers for HHV-6 quantitative RT-PCR analysis**	
U83 Forward	TATGTAGTTCCCCCGATGCG
U83 Reverse	TCTGTTTTCCCAGGTACGGC
U86 Forward	TGCGTTGTTAGAGAGCCCAT
U86 Reverse	GGGAGGGTGGCTACAAAAGA
U89 Forward	ACTTGAGTTGCGGTTGTTGC
U89 Reverse	GGGCATAACCAAACATTGCCA
U90 Forward	CCAGCTAAAGTTTTCAACTCTCCA
U90 Reverse	GTGATACATCCTTCGATGACCTGA
P41 Forward	CCTGTTTTGATGCCAACGCA
P41 Reverse	AAAGCACGTTGTTGACGGTG
